# Drug-resistant *Salmonella* Typhi induced kidney injury with rhabdomyolysis: A case report, and literature review

**DOI:** 10.1016/j.idcr.2021.e01103

**Published:** 2021-03-31

**Authors:** Fateen Ata, Sandy Kamil Kamal Marzouk, Ammara Bint I. Bilal, Adeel Ahmed Khan, Ezzedin Ibrahim, Mohammed Taha Mahmood Almadani

**Affiliations:** a Department of Internal Medicine, Hamad General Hospital, Hamad Medical Corporation, Doha, Qatar; b College of Medicine, Qatar University, Qatar; c Department of Radiology, Hamad General Hospital, Hamad Medical Corporation, Doha, Qatar; d Department of Internal Medicine, Al Wakrah Hospital, Hamad Medical Corporation, Doha, Qatar

**Keywords:** *Salmonella*, Enteric fever, Rhabdomyolysis, Acute kidney injury

## Abstract

*Salmonella* Typhi (ST) is a rod-shaped gram-negative bacterium that infects humans via contaminated food or water. Humans are the only reservoirs for the bacteria. Infection usually manifests as fever, abdominal pain, and diarrhea. However, complications such as intestinal perforation, hepatosplenomegaly, and renal injury can occasionally occur. Acute kidney injury (AKI) is associated more commonly with ST than other bacteria causing gastrointestinal (GI) infections. The exact pathophysiology of AKI in ST infection is unknown. One of the rarely reported mechanisms is rhabdomyolysis. We present a similar case of resistant ST infection leading to AKI with rhabdomyolysis. This article also contains an extensive literature reviews of all reported ST bacteremia cases with AKI secondary to rhabdomyolysis.

## Highlights

• AKI secondary to *Salmonella* Typhi can present with rhabdomyolysis.
• Patients may require temporary dialysis with a prolonged hospital stay.
• The prognosis of ST induced rhabdomyolysis is excellent.
• We present an extensive literature review of all reported ST bacteremia cases with AKI secondary to rhabdomyolysis.

## Introduction

*Salmonella* is estimated to cause more than 93.8 million gastroenteritis cases per annum globally, with 155,000 deaths [1]. It is a rod-shaped gram-negative bacterium of the Enterobacteriaceae family. Amidst around 2500 serotypes of *Salmonella*, only the typhoidal and non-typhoidal serotypes cause infection in humans. *Salmonella* infection may have different clinical presentations. The elderly, children, and immunocompromised patients are prone to develop severe disease. The most common manifestation of salmonellosis is gastroenteritis, presenting clinically with fever, diarrhea, and abdominal pain 12–72 h after infection. This accounts for 70 % of all diagnosed cases. Other clinical presentations are enteric fever (typhoid fever), systemic infection with complications such as gastrointestinal bleeding and perforation, pancreatitis, and AKI [2].

AKI is considerably more common with *Salmonella* Typhi infection (36 %) compared to other GI infections (5%) [3]. One of the mechanisms observed is rhabdomyolysis, reported a few times in the literature [[4], [5], [6], [7], [8], [9], [10], [11], [12], [13], [14], [15], [16], [17], [18], [19]]. Rhabdomyolysis is defined as an injury to the skeletal muscle, resulting in leakage of intracellular contents from myocytes into the plasma [3]. The exact mechanism behind *Salmonella* induced AKI, apart from pre-renal AKI secondary to dehydration, is unknown. One study hypothesized that volume depletion from severe gastroenteritis causes acidic urine, which potentiates the nephrotoxicity of myoglobin and the precipitation of uric acid in the renal tubules, leading to acute renal failure [9]. Other rare causes of AKI in salmonellosis include glomerulonephritis, acute tubular necrosis, and interstitial nephritis [9]. Although there is a higher prevalence of AKI in ST infection, the aspect is still mostly unexplored. We present a case and the most extensive literature review on ST bacteremia induced AKI with rhabdomyolysis.

## Case report

A 39-year-old Pakistani male presented to the hospital with diarrhea and progressively worsening fatigue for ten days. Diarrhea was watery and did not contain any blood. He also complained of decreased appetite, pain while passing urine, and a reduction in the amount of urine for five days. There was no history of fever, abdominal pain, nausea, vomiting, cough, skin rashes, or altered sensorium. The patient had no sick contacts and no history of eating food from outside. The patient was a known case of type 2 diabetes mellitus for two years, was stabilized on metformin 1000 mg twice daily, and was not taking any other medication. He had a recent history of travel to Pakistan, and his symptoms developed during his return flight to Qatar.

On physical examination, the patient was febrile (38 °C) and tachycardiac (104 beats per minute) with normal blood pressure and respiratory rate. The rest of the physical examination was non-significant.

Initial laboratory workup revealed pancytopenia, raised C-reactive protein (CRP), deranged liver enzymes, and elevated creatinine (830) (Table 1). Urinalysis did not show any significant blood cells or casts. Further workup revealed raised creatine kinase (CK) and myoglobin, giving an impression of rhabdomyolysis (Table 1). There was no proteinuria, making diabetic nephropathy unlikely. An ultrasound of the urinary tract revealed bulky kidneys with normal echogenicity, with no features of chronic kidney injury or obstruction. Fluid resuscitation was initiated considering pre-renal AKI. The patient was also empirically started on intravenous (IV) Ceftriaxone 2 g daily, given the possibility of an infectious trigger. The stool sample was negative for Clostridium Difficile toxin, ova, or parasites. There was no bacterial growth in urine and stool samples; however, blood cultures grew *Salmonella* Typhi. Given his recent travel to Pakistan, antimicrobial coverage was escalated to renal adjusted dose of Meropenem (0.5 g every 12 h), considering the possibility of a resistant strain. Subsequently, the sensitivity results revealed a multidrug-resistant *Salmonella* Typhi (including ceftriaxone and azithromycin).

**Table 1:** Laboratory investigations of the patient.

Investigations	Results	Reference range
**Hgb**	12.2 gm/dL	13 – 17 gm/dL
**WBC**	3.8 × 10^3/μL	4 – 10 × 10^3/μL
**Platelets**	28 × 10^3/μL	150−400 × 10^3/μL
**C reactive protein**	38 mg/L	0 – 5 mg/L
**Urea**	31.1 mmol/L	3.2–7.4 mmol/L
**Creatinine**	830 μmol/L	64 – 110 μmol/L
**Myoglobin**	744 ng/mL	28 – 72 ng/mL
**Creatine Kinase**	1,201 U/L	30 – 200 U/L
**AST**	189 U/L	5 – 34 U/L
**ALT**	347 U/L	5 – 34 U/L
**Serum Sodium**	121 mmol/L	133 – 146 mmol/L
**Urine protein**	1140.90 mg/mmol	< = 22.60 mg/mmol
**Urine urea**	24 mmol/L	NA
**Urinalysis**	2 RBC, 3 WBC, No casts	NA

**Fig. 1:**
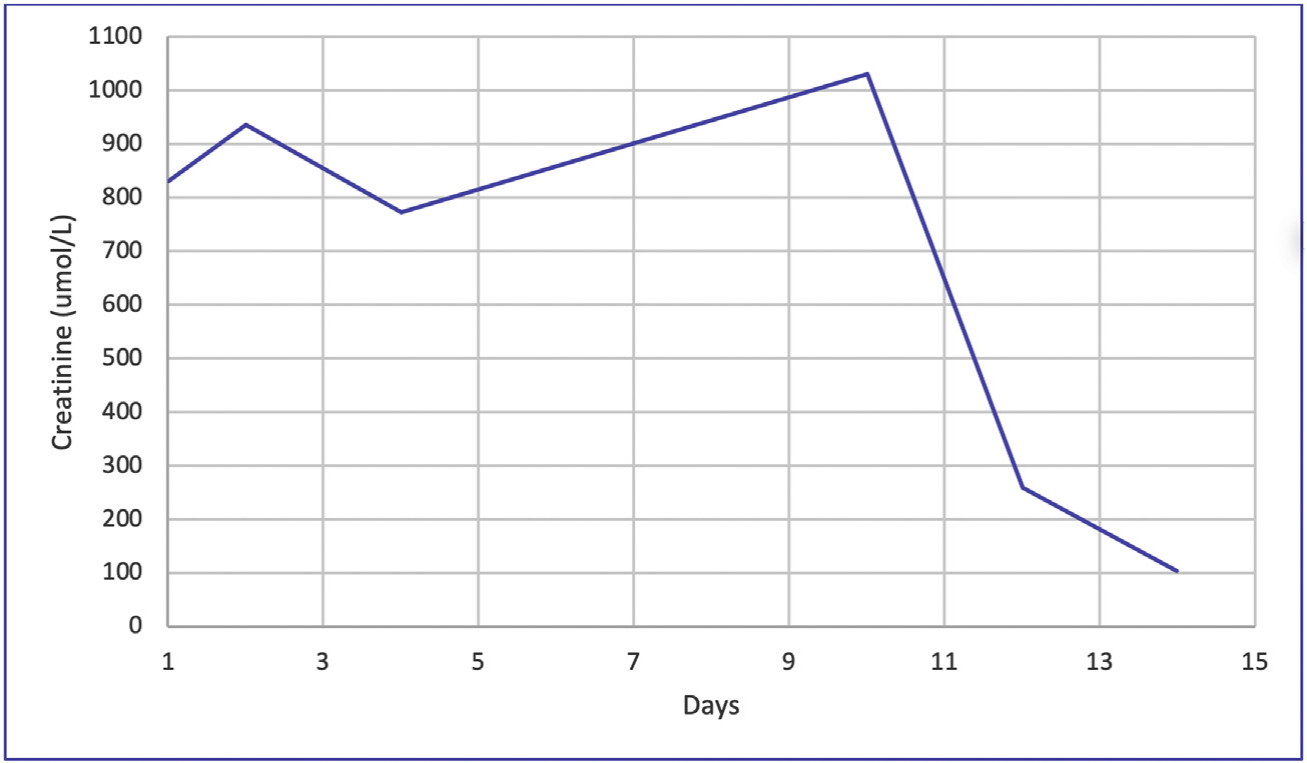
The trend of patient’s creatinine from admission to discharge.

Over the next days, the patient’s creatinine kept rising (Fig. 1), and he became oliguric. His symptoms had mildly improved with a reduction in diarrhea frequency and improvement in his appetite. On day 3, hemodialysis (HD) was initiated due to persistent oliguria and worsening renal parameters despite fluid resuscitation. The patient underwent 4 HD sessions over the next ten days with remarkable creatinine improvement (Fig. 1). On day seven, the patient began to produce urine, which reached up to 2 L per day by day 10. Creatinine normalized by day ten, and repeated blood cultures came negative. The patient took antimicrobials for 14 days (including one day of ceftriaxone and 13 days of meropenem) and was subsequently discharged asymptomatic with normal renal function.

## Discussion

Although more common in resource-limited countries, enteric fever still carries a global health burden [20]. Without treatment, mortality can reach up to 30 percent [20]. Other than classic symptoms of enteric fever such as abdominal pain, anorexia, and diarrhea, ST infection does have atypical presentations. These include neurological symptoms (ranging from headaches to acute psychosis), musculoskeletal (arthralgias, arthritis), hepatobiliary, cardiovascular, and pulmonary manifestations [21]. Additionally, acute kidney injury is also associated with ST infection. The data regarding the mechanism of AKI in ST infection is limited to case reports and one retrospective data review [3]. The retrospective study describes 100 patients with gastrointestinal infections, including 44 with ST infection. Among the cohort, the mean CK level was 126 ± 327 in the ST group, and 167 ± 427 in non-ST infections, and the difference was not statistically significant. The authors concluded that the mechanism of AKI in ST infection might not be explained by dehydration or rhabdomyolysis. However, multiple case reports describe ST infection-induced AKI with rhabdomyolysis [[4], [5], [6], [7], [8], [9], [10], [11], [12], [13], [14], [15], [16], [17], [18], [19]]. Small sample size can be the possible reason that rhabdomyolysis in ST infection is not reflected in the retrospective study.

Although rhabdomyolysis and acute renal failure were described as a complication of typhoid fever as early as 1977, the precise pathophysiologic mechanisms responsible for rhabdomyolysis with *Salmonella* infection are poorly understood due to its rare occurrence [19]. In the existing literature, proposed mechanisms include tissue hypoxia secondary to sepsis, dehydration caused by gastroenteritis, acidosis, electrolyte disturbances, and hypophosphatemia, direct bacterial invasion, activation of lysosomal enzymes, endotoxin release, and altered muscle metabolic capacity caused by low oxidative and glycolytic enzyme activities [13,17].

In an animal study, Friman G et al. explained the mechanism of altered muscle metabolic capacity using rat models infected with ST [22]. The authors found that in the rats’ skeletal muscles, there was a dramatic suppression of oxidative and glycolytic enzyme capacity, in addition to simultaneous upregulation of lysosomal enzyme function. In acute *Salmonella* infection, the oxidative enzyme function was decreased to 65–83 % compared to the control rats. Additionally, the glycolytic enzyme function was only 30–75 % of controls. The authors concluded that *Salmonella* induces rhabdomyolysis by reducing the enzymatic function required to perform a short-time high-intensity exercise and prolonged endurance efforts [22].

We performed an extensive literature review regarding ST gastrointestinal infection-induced rhabdomyolysis. All the articles in English (*N* = 16) were included in the analysis, and data extracted and tabulated (Table 2). A total of 19 cases are reported. Majority of the patients are adult males (*N* = 15). Although ST infection is more prevalent in children, interestingly enough, all the reported cases of ST induced rhabdomyolysis are adults. This may indicate relative protection of the younger population from this complication. The median age of the patients is 50 (32–58) years. All the patients have microbiological evidence of ST infection with AKI. Patients have variable levels of CK (median 6341) ranging from 1124 to 64000 U/L. Our literature review reveals that 6 (31.5 %) patients required HD. The requirement of HD does not seem to correlate with the severity of rhabdomyolysis as it was observed in patients with CK as low as 2801 U/L. Our patient also had relatively mild rhabdomyolysis, yet he required multiple HD sessions. The median length of stay (LOS) is 14 days (10–21). The length of hospital stay in our patient is reflective of the median LOS. Although most patients had a prolonged hospital stay, 100 percent were discharged with a normal renal parameter.

**Table 2:** Literature review of previously reported cases of *Salmonella* Typhi induced rhabdomyolysis.

N	Author	Year	Gender	Age	CPK	HD	Ams started within 24 h	LOS	Outcome
1	Brncic et al. [13]	2002	F	58	64000	Yes	NA	20	Improved
2	Man et al. [11]	1991	M	55	1966	No	NA	14	Improved
3	Rheingold et al. [19]	1977	M	32	17000	No	Yes	21	Improved
4	Campistol et al. case 1 [8]	1989	M	43	1800	No	NA	NA	Improved
5	Campistol et al. case 2 [8]	1989	M	51	4300	Yes	NA	NA	Improved
6	Campistol et al. case 3 [8]	1989	M	38	2270	No	NA	NA	Improved
7	Neau et al. case 1 [9]	2000	M	37	1124	No	Yes	7	Improved
8	Neau et al. case 2 [9]	2000	F	73	3008	No	Yes	14	Improved
9	Abdulla et al. case 1 [4]	1993	M	56	2801	Yes	No	NA	Improved
10	Abdulla et al. case 2 [4]	1993	M	50	32000	No	No	21	Improved
11	Sion et al. [7]	1998	M	56	8600	Yes	Yes	14	Improved
12	Lagarde et al. [12]	1989	F	84	24360	Yes	No	20	Improved
13	Retornaz et al. [18]	1999	M	58	2140	No	NA	12	Improved
14	Al Shamkhani et al. [6]	2015	M	28	3408	No	NA	5	Improved
15	Al-aqeeda et al. [14]	2009	M	34	6341	No	Yes	NA	Improved
16	Dakdouki et al. [10]	2003	M	26	20367	No	NA	28	Improved
17	Fisk et al. [17]	2004	M	25	31410	No	Yes	8	Improved
18	Non et al. [16]	2015	F	21	83350	No	Yes	NA	Improved
19	Jhawar et al. [15]	2012	M	64	9473	Yes	NA	29	Improved
20	Our case	2021	M	39	1201	Yes	Yes	15	Improved

Ams: Antimicrobials, NA: Not available.

ST infection-induced AKI can be severe, and it may appear to have a devastating effect on the renal system initially. With prompt antimicrobial treatment and a timely renal replacement, complete recovery is the most likely outcome.

## Conclusion

This report highlights the possibility of acute kidney injury secondary to rhabdomyolysis in patients with *Salmonella* Typhi infection. Early recognition leads to timely fluid resuscitation and dialysis when needed, resulting in complete renal recovery. More extensive studies are required to understand better the pathophysiology behind ST induced AKI.

## Funding

This article did not receive any funding.

## Consent

Written informed consent for submitting this case report was provided by the patient.

## Ethical approval

Ethical approval was obtained from Medical Research Centre MRC Qatar before submission of this manuscript (MRC-04-21-087).

## Author contribution

**FA**: conceptualization, methodology, literature review, data collection, manuscript writing, critical review and revisions in the manuscript.

**SM**: literature review and manuscript writing.

**AB**: literature review and manuscript writing.

**AK**: literature review and manuscript writing.

**EI**: Data collection, literature review and manuscript writing.

**MA**: literature review and manuscript writing.

**All authors**: final approval of the version to be published.

## Declaration of Competing Interest

The authors report no declarations of interest.
